# The Alpha-defensin Test for Periprosthetic Joint Infection Outperforms the Leukocyte Esterase Test Strip

**DOI:** 10.1007/s11999-014-3722-7

**Published:** 2014-06-19

**Authors:** Carl Deirmengian, Keith Kardos, Patrick Kilmartin, Alexander Cameron, Kevin Schiller, Robert E. Booth, Javad Parvizi

**Affiliations:** 1 CD Diagnostics Inc, Lankenau Institute for Medical Research, The Rothman Institute, 100 Lancaster Avenue, MOB 456, Wynnewood, PA 19096 USA; 2 The Rothman Institute, Thomas Jefferson University, Philadelphia, PA USA; 3 Aria 3B Orthopaedic Specialists, Philadelphia, PA USA

## Abstract

**Background:**

Synovial fluid biomarkers have demonstrated diagnostic accuracy surpassing the currently used diagnostic tests for periprosthetic joint infection (PJI).

**Questions/purposes:**

The purpose of this study is to directly compare the sensitivity and specificity of the synovial fluid α-defensin immunoassay to the leukocyte esterase (LE) colorimetric test strip.

**Methods:**

Synovial fluid was collected from 46 patients meeting the inclusion criteria of this prospective diagnostic study. Synovial fluid samples were tested with both a novel synovial-fluid-optimized immunoassay for α-defensin and the LE colorimetric test strip. The Musculoskeletal Infection Society (MSIS) definition was used to classify 23 periprosthetic infections and 23 aseptic failures; this classification was used as the standard against which the two diagnostic tests were compared.

**Results:**

The synovial fluid α-defensin immunoassay correctly predicted the MSIS classification of all patients in the study, demonstrating a sensitivity and specificity of 100% for the diagnosis of PJI. The α-defensin assay could be read for all samples, including those with blood in the synovial fluid. The leukocyte esterase test strip could not be interpreted in eight of 46 samples (17%) as a result of blood interference. Analysis of the LE strips that could be interpreted yielded a sensitivity of 69% and a specificity of 100%.

**Conclusions:**

The synovial fluid α-defensin immunoassay outperformed the LE colorimetric test strip in this study and provided reliable results even when the LE test strip failed as a result of blood interference. The simple analytic results provided by the α-defensin immunoassay, compared with the more complex and interpretive nature of both the MSIS criteria and LE colorimetric test strip, make it a highly attractive diagnostic tool.

**Level of Evidence:**

Level II, diagnostic study. See Guidelines for Authors for a complete description of levels of evidence.

## Introduction

Periprosthetic joint infection (PJI) is a devastating complication that may occur after joint arthroplasty with major health and economic consequences. Unfortunately, the diagnosis of PJI, which is critical to a timely diagnosis and surgical decision-making, remains a confusing and difficult task. The Musculoskeletal Infection Society (MSIS) has recognized this shortcoming in diagnosis and has offered a definition for PJI that considers clinical findings, several laboratory tests, and tissue histology [9]. Recent efforts to identify simpler and more accurate tools for diagnosing PJI have focused on biomarkers in the synovial fluid [1, 7, 10]. In the field of diagnostics, the term “biomarker” generally refers to a biologically relevant molecule that can be objectively evaluated to indicate a disease or biologic state. Both cytokines and proteins with antimicrobial function have been observed to be elevated in the synovial fluid of patients with an infected joint arthroplasty [2, 5, 7], and many biomarkers have demonstrated diagnostic capabilities better than those of currently used tests [2, 7]. In a recent evaluation of 16 promising synovial fluid biomarkers for PJI, five antimicrobial protein biomarkers were demonstrated to match the MSIS definition of PJI in all 95 study patients [2].

The α-defensin test is an immunoassay that measures the concentration of the α-defensin peptide in human synovial fluid. α-Defensin is an antimicrobial peptide that is secreted into the synovial fluid by human cells in response to pathogenic presence [4]. It then integrates into the pathogen’s cell membrane and causes rapid killing of the pathogen, thus providing antimicrobial support to the immune system [8]. We previously demonstrated that a synovial fluid α-defensin immunoassay exhibited high accuracy for diagnosing PJI, accurately predicting the MSIS classification of all study patients [2].

The leukocyte esterase reagent (LER) test strip is an enzymatic test designed for use in urinalysis and estimates the leukocyte count in urine. When urine is placed on the reagent pad, a detergent lyses the urine leukocytes and releases esterases, capable of catalyzing a reaction that leads to formation of a violet dye. Although the LER test was not developed for synovial fluid and is not a specific immunoassay, it has been found to be useful for the diagnosis of PJI [10, 12] and is being used clinically for that purpose [12].

Synovial fluid biomarker research has revealed many potential biomarkers for PJI; however, none of these immunoassay biomarker tests has been directly compared with the LER test to assess for optimal diagnostic characteristics. Both our earlier work [2] and this study are based on a synovial fluid archive that our center has maintained since 2009. Although the majority of patient samples in this study were also included in the previous work, the current study independently retested all patient samples using an immunoassay for synovial fluid α-defensin and directly evaluates the results in comparison to the LER test strip. The purpose of this study is to compare the sensitivity and specificity of the α-defensin immunoassay with that of the LER colorimetric test strip in diagnosing PJI in a selected set of patient samples.

## Patients and Methods

### Study Design

The study was approved by the institutional review board. The current study includes synovial fluid samples from an institutional archive, almost all of which were previously included in a previously published study [2]. The synovial fluid samples included in this study were independently tested for α-defensin for the purposes of this study.

Given the lack of published data regarding the α-defensin immunoassay, we did not perform a power analysis before the study. As part of a biomarker screening program initiated in 2009, our institution archives and prospectively annotates synovial fluid samples from the patients of adult arthroplasty surgeons. Patient inclusion in the current study required (1) an evaluation for possible infection of a THA or TKA; (2) sufficiently annotated clinical and laboratory data for classification by the MSIS criteria for PJI; and (3) sufficient synovial fluid for study methods. Patients receiving antibiotics before aspirations and patients having the diagnosis of a systemic inflammatory disease were not excluded from this study. Patients meeting the study’s inclusion criteria were prospectively evaluated and classified as infected or aseptic as defined by the MSIS (Table 1). Additionally, sex, age, joint, surgical findings, and isolated organism were recorded when pertinent.

**Table 1:** MSIS Workgroup standard definition for PJI

One of the following must be met for diagnosis of PJI:
1. A sinus tract communicating with the prosthesis;
2. A pathogen is isolated by culture from two separate tissue
or fluid samples obtained from the affected prosthetic joint;
3. Four of the following six criteria exist:
a. Elevated ESR and CRP (ESR > 30 mm/hr; CRP > 10 mg/L),
b. Elevated synovial fluid WBC count (> 3000),
c. Elevated synovial fluid neutrophil percentage (> 65%),
d. Presence of purulence in the affected joint,
e. Isolation of a microorganism in one periprosthetic tissue or fluid culture,
f. Greater than 5 neutrophils per high-powered field in 5 high-powered fields observed from histological analysis of periprosthetic tissue at 400 times magnification.

MSIS = Musculoskeletal Infection Society; PJI = periprosthetic joint infection; ESR = erythrocyte sedimentation rate; CRP = C-reactive protein; WBC = white blood cells.

### Patients

From January 2012 to August 2012, 23 patients classified as having an aseptic joint effusion were identified who met the inclusion criteria of the study, requiring an MSIS classification and sufficient remaining synovial fluid for an α-defensin test, and had a documented LER test. No other aseptic patients during the study period met the inclusion criteria. This group included 13 women and 10 men with a mean age of 63 years. There were 21 knees and two hips. The aseptic diagnoses included 17 patients with aseptic loosening, three patients with pain without a mechanical cause, one knee with pseudogout, one hip revised for corrosion at a dual modular junction, and one retained cement spacer. Of the 23 patients with an aseptic diagnosis, four had a comorbid systemic inflammatory disease.

Over the same time period, 13 patients classified as having a PJI were identified who met the inclusion criteria of the study, requiring an MSIS classification, an α-defensin test result, and a LER test result. No other patients with PJI met the inclusion criteria of this study during the study period. To include an equal number of samples with the diagnosis of PJI, we started with the opening date of the study and worked backward through our institutional archive of synovial fluid samples, applying inclusion criteria until an additional 10 consecutive samples were identified. A total of 23 patients with PJI were thus included with 15 women and eight men at a mean age of 67 years. This group included 22 knees and one hip. Eighteen joints were associated with a positive culture, whereas five were culture-negative infections using the MSIS criteria to define them as having an infection.

Organisms included *Staphylococcus epidermidis* (six), *Staphylococcus aureus* (methicillin-sensitive *S aureus* = two; methicillin-resistant *S aureus* = five), *Escherichia coli* (one), *Serratia marcescens* (one), *Corynebacterium striatum* (one), *Streptococcus mutans* (one), and multiorganism (one). Of the 23 patients with PJI, six had a comorbid systemic inflammatory disease, and six patients were started on antibiotic treatment before culture samples were collected.

The MSIS relevant laboratory data were recorded for each group (Table 2).

**Table 2:** MSIS relevant laboratory and clinical findings

Finding	Aseptic (23)	PJI (23)
Sinus	0	3
At least one positive culture	0	18
Mean ESR	24 mm/hr	88 mm/hr
Mean CRP	6 mg/L	109 mg/L
Mean WBC	641	32,043
Mean neutrophil (%)	29%	87%

MSIS = Musculoskeletal Infection Society; PJI = periprosthetic joint infection; ESR = erythrocyte sedimentation rate; CRP = C-reactive protein; WBC = white blood cells.

### Sample Preparation and Biomarker Analysis

Synovial fluid was delivered to the laboratory immediately after aspiration. Aliquots for α-defensin testing were subjected to centrifugation to separate all particulate and cellular material from each synovial fluid sample, and the resulting supernatant was aliquoted and frozen at −80° C. Synovial fluid samples were independently tested for α-defensin for the purposes of this study. Aliquots for the LER tests were tested at the time of sample collection without processing of the synovial fluid.

The immunoassay for synovial fluid human α-defensin 1–3 was generated using reagents from Hycult Biotech (Uden, The Netherlands) and measured in duplicate by standard enzyme-linked immunosorbent assay. The assays were optimized specifically for performance in synovial fluid by scientists with specific training in immunoassay development (AC, KS, KK, PK). This optimization included dilution optimization of the synovial fluid to eliminate the effects of varying viscosity between samples. Synovial fluid standards, for consistent assay calibration, were established for the α-defensin immunoassay. The ultimate goal of optimization was to minimize the synovial fluid matrix effect and center the linear performance of the assays on the diagnostic cutoff. The assay for α-defensin was optimized to operate at a cutoff value of 5.2 mg/L, which was used to provide a diagnosis of aseptic disease or PJI. The choice of this cutoff value for α-defensin was based on previously published studies [2] in combination with unpublished studies at our institution. This cutoff value was not optimized by applying receiver operating characteristic curves specifically for this study. However, the majority of the patients in this study were included in a larger patient cohort that was used to choose the cutoff of 5.2 mg/L. The lower limit of α-defensin assay detection was 1.56 mg/L. Any lower values were set at this value for the purposes of analysis.

The LER test used in this study was the Chemstrip 7 urine test strip (Roche Diagnostics, Indianapolis, IN, USA). We defined a 2+ result to be positive for PJI. The LE test strip is a colorimetric method that is based on the hydrolysis of an active substrate by the leukocyte esterase that reacts with a diazonium salt to form a purple color. The method yields a semiquantitative result that is related to the total number of leukocytes that are present in the sample. The results for the test strip are interpreted as negative (white), trace (slightly purple), + (light purple), or ++ (dark purple). Laboratory personnel were trained to properly interpret the test strip results. If blood or debris was judged to impede the ability to interpret the result, then the result was categorized as “unreadable.”

### Data Analysis

The results of all synovial fluid testing results were compared between infected and aseptic joints based on the MSIS definition. The sensitivity and specificity of each assay were calculated along with corresponding confidence intervals. Additionally, the percentage of unreadable results was calculated for each test. Fisher’s exact test was used to calculate statistically significant differences between the test’s overall number of correctly diagnosed patients and the overall number of unreadable tests.

## Results

The synovial fluid α-defensin immunoassay correctly diagnosed 100% of patients in this study, whereas the LER test was able to correctly diagnose 33 of 46 patients (78%) (p < 0.001). The LER test resulted in five false-negative results, no false-positive results, and eight uninterpretable results.

**Fig. 1:**
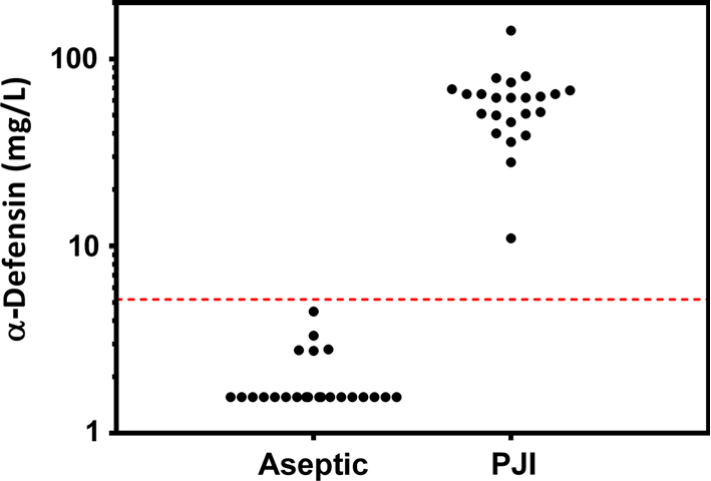
α-Defensin concentrations in patients with aseptic disease versus PJI are demonstrated. Note that concentrations are depicted on a log scale.

The synovial fluid α-defensin immunoassay demonstrated a sensitivity and specificity of 100% (95% confidence interval [CI], 85.05%–100%) for the diagnosis of PJI in this study (Fig. 1). The average α-defensin concentration among infected samples was 59.60 mg/L, which was 31-fold higher than the average level among aseptic samples (1.92 mg/L). Eighteen of 23 aseptic samples (78%) had undetectable levels of α-defensin, and the concentration was therefore set to the minimum detectable concentration of 1.56 mg/L. The α-defensin assay provided a valid result for all samples, including those with blood contamination.

Additionally, although the synovial fluid α-defensin immunoassay was able to provide a valid result for all samples in this study, the LER test was unreadable in eight of 46 joints as a result of blood interference (17%; p < 0.001). Analysis of the remaining LE strips yielded a sensitivity of 68.8% (95% CI, 41.36%–88.87%) and a specificity of 100% (95% CI, 84.43%–100%), resulting from five false-negative results (Table 3).

**Table 3:** Analysis of leukocyte esterase strips

LER result	Aseptic (n = 23)	PJI (n = 23)
2+		11
1+		
Trace	1	
Absent	21	5
Unreadable	1	7

LER = leukocyte esterase reagent; PJI = periprosthetic joint infection.

## Discussion

The diagnosis of PJI is a most critical step in the management of a patient with a painful arthroplasty, because it guides surgical decision-making. However, the currently used laboratory tests for infection are not able to provide a sufficiently accurate diagnosis of PJI. It is for this reason that the MSIS created a definition of PJI [9], including several clinical and laboratory findings that can be combined to achieve a diagnostic accuracy that is better than any individual test. Synovial fluid biomarkers have demonstrated great promise to provide a highly accurate diagnosis of PJI [1, 5, 7]. We previously demonstrated that a synovial fluid α-defensin immunoassay exhibited high accuracy for diagnosing PJI, accurately predicting the MSIS classification of all study patients [2], but did not directly compare those results with the LER test. Both our earlier work [2] and this study are based on a synovial fluid archive that our center has maintained since 2009. Although the majority of patient samples in this study were also included in the previous work, the current study independently retested all patient samples using an immunoassay for synovial fluid α-defensin and directly evaluates the results in comparison to the LER test strip. The primary purpose of this study was to compare the diagnostic performance of α-defensin and the LER test.

There are several limitations to this study. First, the study includes an equal number of patients with an aseptic diagnosis or PJI. We chose to conduct the study this way to maximize the number of PJIs and achieve a best estimate of the tests’ sensitivities and specificities, which are not dependent on disease prevalence. Because of this unnatural study prevalence, positive and negative predictive values could not be calculated. Ideally, studies evaluating a diagnostic test would include a prevalence of disease similar to that in clinical practice to allow for determination of the test’s predictive value. Second, the LER test strips were interpreted by trained personnel, which may not reproduce users of the test strips in practice. This may cause overestimation of the performance of the LER test strip. Finally, a predominant number of the collected samples was from knees, mostly as a result of the fact that higher fluid volumes were necessary to satisfy the inclusion criteria, potentially limiting the confident transfer of this study’s conclusions to hip arthroplasty.

The appeal of a biomarker immunoassay developed for PJI is that it would provide objective, analytical, and consistent results for all surgeons with no need for test interpretation. In this study, the α-defensin cutoff value of 5.2 mg/L provided complete separation between the aseptic and infected joint arthroplasties without failures resulting from uninterpretable results. To our knowledge, the largest study to date evaluating the diagnostic performance of the leukocyte esterase reagent test was published by Wetters et al. [12]. They included 223 arthroplasties to evaluate the diagnostic characteristics of the LER test strip for the diagnosis of PJI and considered a 1+ or 2+ LER reading as indicating a positive result. The LER test was unreadable for 29% of the 223 arthroplasties tested as a result of debris, blood, or ambiguous results. Of the remaining 158 tests that were readable, the sensitivity ranged from 93% to 100% and the specificity ranged from 77% to 89%, depending on the definition of infection. Parvizi et al. [10] also reported on the diagnostic characteristics of the LER test in a study of 108 knees. When defining a positive LER test as a 1+ or 2+ result, like in the Wetters et al. [12] study, they found a sensitivity of 93.5% and a specificity of 86.7%. When defining a positive LER as a 2+ result, they demonstrated 80.6% sensitivity and 100% specificity. In the current study, we identified 17% of the samples tested by the LER test as unreadable. We considered a 2+ result to be indicative of PJI and, similar to the report by Parvizi et al., found a 100% specificity coupled with a relatively low sensitivity of 69%. However, choosing to include 1+ results, like in the report by Wetters et al. [12], would not have changed our results.

The LER test was developed for and cleared regulatory approval for urinalysis, and its performance and interpretation are predicated on the assumption that urine is being tested [11]. The high viscosity [3] and varied chemical properties of synovial fluid do not parallel urine, so it remains unclear whether the subjective interpretations made by the trained personnel in the larger academic studies described would be translated to all surgeons when using synovial fluid. Second, because the LER test pad causes lysis of leukocytes, the LER test is not measuring secreted esterase activity, but instead is measuring total intracellular and extracellular activity, essentially acting as a proxy to the leukocyte count. Therefore, if the LER test is performing in synovial fluid as intended to perform in urine, the diagnostic characteristics of the test should be reasonably equivalent, but not superior, to those of a synovial fluid leukocyte count. It must also be noted that the LER test carries a warning of potential interference from elevated protein, elevated glucose, and several antibiotics, which may be present in synovial fluid.

The α-defensin test used in this study is based on an immunoassay platform, which is a common technology currently used in medicine [6]. The α-defensin immunoassay was developed and optimized specifically for use in synovial fluid, minimizing any potential chemical and viscosity effects as a result of variations in synovial fluid. Finally, the α-defensin immunoassay only measures extracellular levels of α-defensin, potentially avoiding falsely elevated results from elevated white blood cell counts in the setting of aseptic disease.

This study benefited from consistent application of the MSIS definition of PJI in the classification of all patients and from the inclusion even of patients with potentially confounding conditions (such as systemic inflammatory diseases and antibiotic treatment), which we believe increases the generalizability of our findings to routine clinical practice. We found that the α-defensin immunoassay outperformed the LE colorimetric tests strip in this study and provided reliable results even when the LE test strip failed as a result of blood interference. The simple analytic results provided by the α-defensin immunoassay when compared with the more complex and interpretive nature of both the MSIS criteria and LE colorimetric test strip make it a highly attractive diagnostic tool for clinical use.

## References

[1] Deirmengian C, Hallab N, Tarabishy A, Della Valle C, Jacobs JJ, Lonner J, Booth RE (2010). Synovial fluid biomarkers for periprosthetic infection. Clin Orthop Relat Res..

[2] Deirmengian C, Kardos K, Kilmartin P, Cameron A, Schiller K, Parvizi J. Diagnosing periprosthetic joint infection: has the era of the biomarker arrived? *Clin Orthop Relat Res.* 2014 Mar 4 [Epub ahead of print]. 10.1007/s11999-014-3543-8 PMC4182392 24590839

[3] Fam H, Bryant JT, Kontopoulou M (2007). Rheological properties of synovial fluids. Biorheology..

[4] Ganz T, Selsted ME, Szklarek D, Harwig SS, Daher K, Bainton DF, Lehrer RI (1985). Defensins. Natural peptide antibiotics of human neutrophils. J Clin Invest..

[5] Gollwitzer H, Dombrowski Y, Prodinger PM, Peric M, Summer B, Hapfelmeier A, Saldamli B, Pankow F, von Eisenhart-Rothe R, Imhoff AB, Schauber J, Thomas P, Burgkart R, Banke IJ (2013). Antimicrobial peptides and proinflammatory cytokines in periprosthetic joint infection. J Bone Joint Surg Am..

[6] Hendriks HA, Kortlandt W, Verweij WM (2000). Standardized comparison of processing capacity and efficiency of five new-generation immunoassay analyzers. Clin Chem..

[7] Jacovides CL, Parvizi J, Adeli B, Jung KA (2011). Molecular markers for diagnosis of periprosthetic joint infection. J Arthroplasty..

[8] Lehrer RI, Ganz T (1992). Defensins: endogenous antibiotic peptides from human leukocytes. Ciba Found Symp..

[9] New definition for periprosthetic joint infection. *J Arthroplasty.* 2011;26:1136–1138. 10.1016/j.arth.2011.09.026 22075161

[10] Parvizi J, Jacovides C, Antoci V, Ghanem E (2011). Diagnosis of periprosthetic joint infection: the utility of a simple yet unappreciated enzyme. J Bone Joint Surg Am..

[11] Wenz B, Lampasso JA (1989). Eliminating unnecessary urine microscopy. Results and performance characteristics of an algorithm based on chemical reagent strip testing. Am J Clin Pathol..

[12] Wetters NG, Berend KR, Lombardi AV, Morris MJ, Tucker TL, Della Valle CJ (2012). Leukocyte esterase reagent strips for the rapid diagnosis of periprosthetic joint infection. J Arthroplasty..

